# Assessment of 3D-Printed Tooth Containing Simulated Deep Caries Lesions for Practicing Selective Caries Removal: A Pilot Study

**DOI:** 10.3390/ijerph20010090

**Published:** 2022-12-21

**Authors:** Piyaphong Panpisut, Patchayaporn Doungkom, Chawal Padunglappisit, Worachate Romalee, Nattira Suksudaj

**Affiliations:** 1 Faculty of Dentistry, Thammasat University, Pathum Thani 12120, Thailand; 2 Thammasat University Research Unit in Dental and Bone Substitute Biomaterials, Thammasat University, Pathum Thani 12120, Thailand; 3 Faculty of Engineering and Technology, Pathumthani University, Pathum Thani 12000, Thailand; 4 College of Dentistry, National Yang Ming Chiao Tung University, Taipei 112, Taiwan

**Keywords:** dental caries, dental education, dental students, operative dentistry, 3D printing, selective caries removal

## Abstract

A standard model for practicing caries removal skills is needed to support learners in managing deep carious lesions. The aim of the current study was to prepare 3D-printed teeth with added simulated carious layers and a pulpal structure. A first permanent mandibular containing occlusal (Class I) or proximal (Class II) cavities was printed. The teeth were then filled with wax and resin-modified glass ionomer cements mixed with a color modifier to simulate pulp and deep caries, respectively. Undergraduate dental students (*n* = 61) were asked to remove the caries using the selective caries removal (SCR) technique on the teeth. The students then completed a self-administered questionnaire to rate their caries removal experiences. One instructor then assessed the prepared teeth. Overall, the students provided positive feedback on the use of 3D-printed teeth; 72.1% agreed that the printed teeth provided a realistic model for practicing the SCR technique, 75.4% indicated that the new teeth were the appropriate choice for practicing the SCR technique, and 86.9% agreed that 3D-printed teeth should be used before treating real patients. More than half of the students had satisfactory outcomes in terms of the depth and caries removal aspects of the cavity preparation. These findings suggest that the developed 3D-printed teeth can potentially be adopted to practice caries removals in preclinical dental education.

## 1. Introduction

Untreated dental caries is the most common chronic and preventable disease affecting people globally [1]. The continuation of an active caries eventually leads to the formation of a cavitated lesion, which requires restorative treatment. Dentists should employ the selective caries removal (SCR) technique when treating deep cavities to reduce the risk of pulpal complications. In the SCR technique, firm and leathery dentin or affected dentin is left over the pulp, but a substantial sound tooth should be obtained at the peripheral area to ensure the adequate sealing of the restoration. The SCR technique has shown desirable long-term outcomes and is more cost effective compared with the traditional total caries removal technique [2]. Several dental schools have, therefore, adopted the SCR technique into both preclinical and clinical curricula for undergraduate students [3].

In various countries, dental students usually practice the SCR technique in severely damaged extracted teeth. However, the extension of caries lesions in such teeth may be too aggressive, which may make the teeth unsuitable for practice. This could subsequently reduce confidence and increase the stress levels of dental students during the treatment of deep caries lesions in real patients [4]. Hence, a standard tooth model containing sufficiently deep caries lesions is needed for preclinical practice.

The advancement of scanning technologies and 3D printing innovation has brought the opportunity to produce a variety of simulated tooth models for caries removal practice [5,6,7]. Three-dimensional printed teeth can mimic complicated tooth structures and can be more realistic and accurate than typodont teeth. Researchers have introduced the use of 3D-printed teeth with anatomical details, such as occlusal anatomy and dental pulp, for training cavity preparation skills [5,7,8,9,10,11]. The results of these studies show that dental students perceive the use of 3D-printed teeth with anatomical details to be satisfactory. Additionally, the use of 3D-printed teeth enhances the clinical skills required to clinically treat real patients [10].

The main challenge in preparing the teeth is mimicking the appearance of deep caries lesions over the pulp chamber. The fidelity and accuracy of the teeth, which contain both deep caries lesions and a pulpal structure, are expected to encourage positive learning experiences among students. Therefore, the aim of this study was to prepare 3D-printed teeth with anatomical details and simulated carious lesions for preclinical training using the selective caries removal technique. We also examined the perception of the students toward the use of 3D-printed teeth for caries removal practice.

## 2. Materials and Methods

### 2.1. The Preparation of the Tooth Model

**Figure 1:**
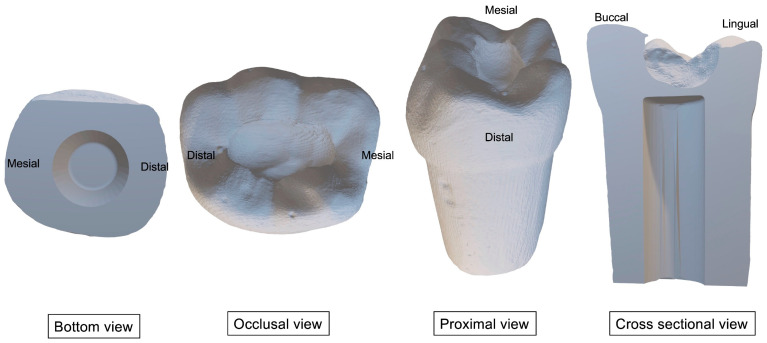
3D images of the permanent lower-left first molar with the occlusal cavity.

**Figure 2:**
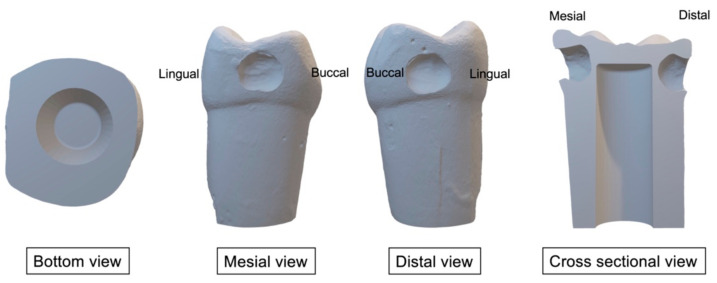
3D images of the permanent lower-left first molar with the proximal cavity.

Table 1 presents the materials that we used to prepare the 3D-printed teeth and caries layers. Firstly, we prepared occlusal (Class I) and proximal (Class II) cavities on the standard permanent left first molar plastic tooth. We scanned the prepared teeth using an intraoral scanner (CEREC Primescan AC, DENSPLY Sirona, Charlotte, North Carolina, USA) to produce two STL files of the tooth containing occlusal (Class I) (Figure 1) or proximal (Class II) (Figure 2) cavities, respectively. We modified the files using SolidWorks (Dassault Systèmes SolidWorks Corporation, Waltham, MA, USA) to produce space within the tooth to mimic the dental pulp chamber. We then printed the teeth using 3D printer resin (optiprint model, Dentona AG, Dortmund, Germany) and a 3D printing machine (ASIGA MAX UV, Asiga, NSW, Australia). Then, we washed the printed teeth with 97% isopropanol and cured them in a UV chamber for 30 min.

**Figure 3:**
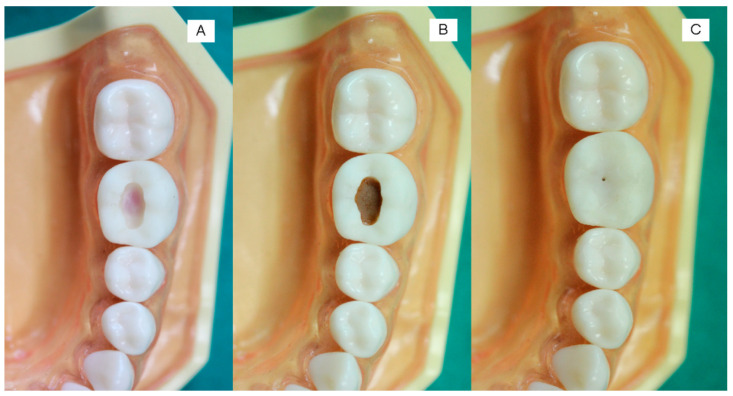
The steps to create a Class I cavity. (**A**) We filled the tooth with red-colored dental wax from the bottom side to mimic the dental pulp. (**B**) We filled the tooth with RMGIC mixed with a color modifier for ~3 mm to mimic a caries lesion. The light-curing time was 5 s. (**C**) We filled the final layer with a nanofilled resin composite. We created the small opening to guide the students to the location of cavitated caries.

**Figure 4:**
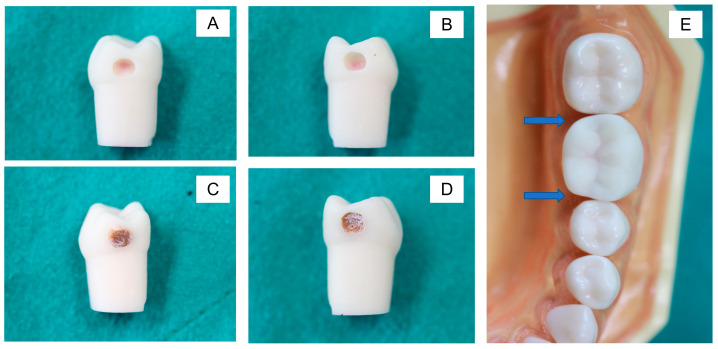
(**A**,**B**) Class II cavities in proximal surfaces. (**C**,**D**) We filled the cavities with RMGIC mixed with the color modifier. (**E**) We placed the tooth in a model. A slight dark shadow (arrows) can be observed underneath the marginal ridges, which could indicate the location of caries lesions.

The printed teeth were filled with dental wax from the bottom to mimic the appearance of dental pulp (Figure 3A and Figure 4A). Then, the caries layer was placed on the deepest part of the occlusal and proximal cavities. The simulated caries layer was prepared with resin-modified glass ionomer cements using a powder-to-liquid ratio of 1:3. We added the color modifier (~1 wt%) to the resin-modified glass ionomer cement (RMGIC) to mimic the brown/yellowish appearance of a caries lesion. The RMGIC was placed in the deepest part of the cavity (thickness ~3 mm) and light-cured using a light-emitting diode (LED) curing unit for 5 s (Figure 3B and Figure 4C,D). For the occlusal cavity, we covered the simulated caries layer with a nanofilled resin composite for 1–2 mm (Figure 3C). For the proximal surface, we left the simulated caries uncovered at the proximal side to produce the shadow appearance on the marginal ridge (Figure 4E).

### 2.2. Data Collection

The Ethical Review Subcommittee for Research Involving Human Research Subjects of Thammasat University, Thailand, approved the current study (ID: 039/2564; approval date: 27 April 2021). The students who participated in this study voluntarily signed informed consent forms. In total, 61 (88%) fifth-year clinical students were involved in this study.

We developed a self-administered questionnaire to obtain the students’ opinions on using the 3D-printed tooth. The questionnaire included 10 items with a 5-point rating scale (1 = strongly disagree, 2 = disagree, 3 = neutral, 4 = agree, 5 = strongly agree) and one open-ended question that dental schools in Thailand commonly employ (Table 2). Three experts validated the questionnaire’s content validity (between 0.6 and 1.0) based on the item objective congruence (IOC) index.

We fixed the teeth within a dental model (Nissin Dental Products Inc, Kyoto, Japan) in a phantom head. The students received two teeth containing occlusal (Class I) and proximal lesions (Class II). We instructed the students to perform the minimally selective caries removal technique with standard instruments and high-speed and low-speed burs. The students had 20 min to prepare two teeth with occlusal and proximal lesions. We provided no additional feedback during the task. Then, the students were required to complete the questionnaire. The final question allowed the students to provide text responses to express their opinion, feedback, or comments regarding their use of the printed teeth. One evaluator (Piyaphong Panpisut) rated the students’ performance. We then used Microsoft Excel version for macOS v. 16.66.1 (Microsoft, Redmond, Washington, DC, USA) to perform descriptive statistical analyses. Additionally, we statistically determined the differences between the students’ performance of each component in the caries removal for the Class I and II cavities using a chi-square test (Prism 9 for macOS, GraphPad Software, LLC., San Diego, CA, USA). We set the significance level at *p* = 0.05.

## 3. Results

**Figure 5:**
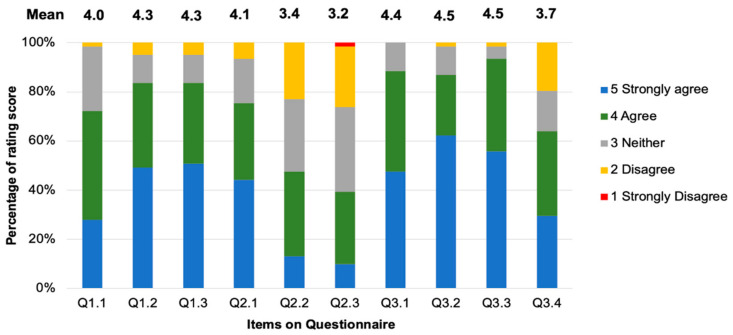
Percentage of each answer for each item on the questionnaire assessing the use of the 3D-printed teeth.

Q1.1–1.3 assessed the students’ opinions on using 3D-printed teeth compared with a standard plastic tooth. For Q1.1, 72.1% of the students stated that they “Agree” or “Strongly Agree” (mean ± SD = 4.0 ± 0.79) (Figure 5) that the 3D-printed teeth with added caries were more realistic than the plastic tooth that was previously used to practice the SCR technique. However, 26% and 2% responded with “Neutral” or “Disagree” for this question, respectively. For Q1.2, 84% of the students stated that they “Strongly Agree” or “Agree” that the 3D-printed teeth with caries lesions should be used to practice the SCR technique instead of the standard plastic teeth (mean ± SD = 4.3 ± 0.86). In comparison, 16% and 5% of the students responded with “Neutral” and “Disagree”, respectively. The last question in this section asked if the 3D-printed teeth were easy to handle compared with plastic teeth. In total, 84% of the students responded with “Strongly Agree” or “Agree” (mean ± SD = 4.3 ± 0.86), whilst 16% of them responded with either “Neutral” or “Disagree”.

The second part of the questionnaire (Q2.1–2.3) assessed the students’ opinions on using a 3D-printed tooth compared with an extracted tooth, which they used during their preclinical training. For Q2.1 (mean ± SD = 4.1 ± 0.94), 75% of students stated that they “Strongly Agree” or “Agree” that the 3D-printed teeth with added caries are the more appropriate choice for practicing the SCR technique compared with extracted teeth. In contrast, 25% of them stated “Neutral” or “Disagree”. However, 53% of the students responded with “Neutral” or “Disagree” when asked if the 3D-printed teeth provided more realistic tactile feedback during cavity preparation compared with extracted teeth (mean ± SD = 3.4 ± 0.98). Conversely, 48% responded with “Strongly Agree” or “Agree”. The last question in the section concerned the tactile feeling during the caries removal step. Only 39% of the students stated that they “Strongly Agree” or “Agree” that the 3D-printed teeth provided a realistic tactile sensation during the caries removal step that was similar to that provided by extracted teeth (mean ± SD = 3.2 ± 0.98), whereas 59% of the students responded with “Neutral” or “Disagree”.

The third section of the questionnaire (Q3.1–3.3) concerned the use of 3D-printed teeth with added caries lesions to increase the students’ skills and motivation to use the SCR technique. In total, 89% of the students stated that they “Strongly Agree” or “Agree” that the use of 3D-printed teeth could increase their motivation to practice their skills with the SCR technique compared with plastic and extracted teeth (mean ± SD = 4.4 ± 0.68). However, 12% responded with “Neutral” for this question. Additionally, 87% stated that they “Strongly Agree” or “Agree” that they should have used the 3D-printed teeth to practice the SCR technique before treating real patients (mean score ± SD = 4.5 ± 0.77). In contrast, 13% of the students responded with “Neutral” or “Disagree”. Additionally, 93% of the students stated that they “Strongly Agree” or “Agree” that using the 3D-printed teeth helped them practice controlling basic fine movements of the fingers (mean ± SD = 4.5 ± 0.67). However, 7% responded with “Neutral” or “Disagree”. The last question asked if the use of 3D-printed teeth with added caries lesions helped them increase their SCR technique skills. The result showed that 64% of the students responded with “Strongly Agree” or “Agree” for this question (mean ± SD = 3.7 ± 1.09), whilst 36% responded with “Neutral” or “Disagree”.

**Figure 6:**
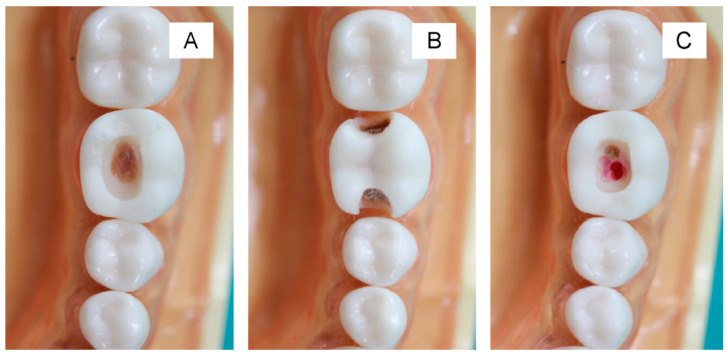
Example of the task completed after cavity preparation. Class I (**A**) and II (**B**) with remaining caries over the pulp to prevent pulp exposure. The peripheral margin of the prepared cavity was in the sound tooth structure. Excessive wax appeared when the preparation was too deep (**C**).

**Figure 7:**
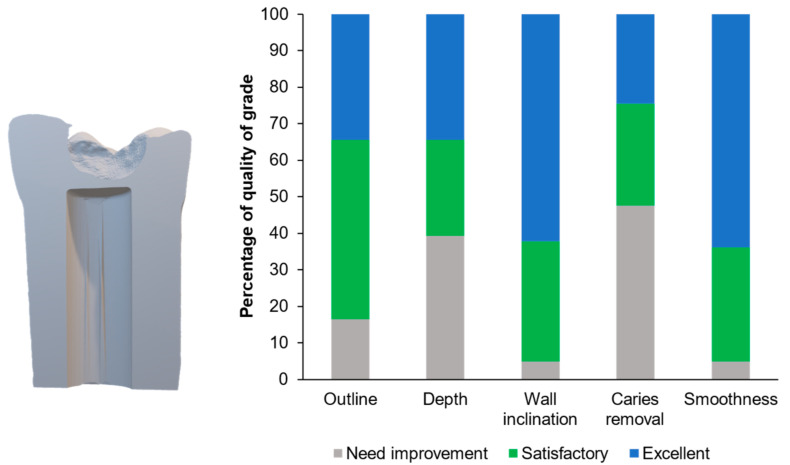
Percentage of grades for each parameter evaluated for the occlusal cavity task.

**Figure 8:**
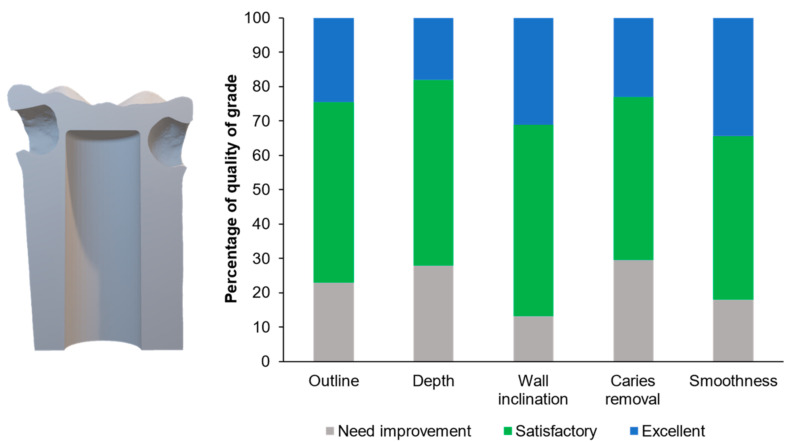
Percentage of grades for each parameter evaluated for the proximal cavity task.

Figure 6 illustrates examples of the students’ cavity preparation outcomes. The evaluator rated 34% and 49% of the prepared teeth as excellent and satisfactory, respectively, according to the outline of the final outcomes (Figure 7). For the depth and caries removal of the prepared teeth, the evaluator rated 61% and 52% of the prepared teeth as satisfactory and excellent, respectively (Figure 7). Additionally, the evaluator rated 95% of the prepared teeth as satisfactory and excellent for the wall inclination and smoothness. For the proximal cavities (Figure 8), the evaluator rated the preparation outline, depth, wall inclination, caries removal, and smoothness as satisfactory and excellent for 70–82% of the prepared teeth (Figure 8). Additionally, we detected significant differences between the proportion of the scores between the Class I and II cavities for depth (*p* = 0.0003), wall inclination (*p* < 0.01), caries removal (*p* = 0.0087), and smoothness (*p* < 0.01). The proportion of the outline performance scores for the Class I and II cavities were comparable (*p* = 0.2575).

The students provided several comments on the use of the 3D-printed teeth. First, the hardness of the enamel layer of the 3D-printed teeth was softer than that of plastic teeth. Second, differentiating caries lesions was difficult, and the students noted that the tactile feeling during caries removal was not similar to what is found in real patients. However, some students stated that the colored caries facilitated caries removal in comparison with the standard model. The 3D-printed teeth were more useful for training than plastic teeth.

## 4. Discussion

This study’s aim was to develop customized, simulated 3D-printed teeth with added caries lesions to practice selective caries removal in preclinical dental education. The 3D-printed teeth used in this study contained a pulpal chamber similar to the teeth with simulated caries lesions in previous studies [5,7]. This structure allows students to be aware of the extent of caries removal in relation to the pulp–dentin complex, which affects the longevity of the treatment. The advantage of the 3D-printed model is that it can be customized to fit with the model currently used for training [5,7,12]. Additionally, the size or location of the cavities on the tooth can be designed to enable deliberate practice in various conditions.

When answering open-ended questions, the dental students indicated that the distinctive-colored caries lesions helped them to perform the caries removal and cavity preparation more effectively than those in the standard model. These results are consistent with a previous study using multicolored 3D-printed teeth that promoted the self-learning of cavity preparation [13]. The use of 3D-printed teeth with added caries lesions can be modified for other cavity types. This may enhance the self-learning skills of students and increase their confidence to practice with real patients [14]. The major limitation of VR techniques is their high cost. Additionally, the students who practice using VR haptic simulators tend to exhibit inferior performance for complex restorative treatment and are less likely to appropriately hold instruments compared with those who practice with a conventional phantom head [15,16]. We found that the students correctly performed the caries removal in the proximal cavities most often after they finished practicing in the occlusal cavity. This may correlate with the concept of experiential learning [17]. Students may have gained concrete experience after practicing the first task. This may have allowed them to self-reflect or recall and conceptualize the selective caries removal technique. The student may have extracted the essence of the technique from the first task to perform a similar task in proximal cavities.

In reality, the extent of a caries removal mainly relies on the differences between the texture of the lesions and that of sound tooth structure. Infected or affected dentin is usually softer than sound dentin and enamel, which aids the clinician in preserving the sound tooth structure in the peripheral area. The limitation of the simulated teeth used in this study is that the hardness of the caries lesions may have been too high, which made it difficult to differentiate using tactile sensation. The students also commented on the similar hardness between the caries lesions and the sound tooth. We produced the caries layer using resin-modified glass ionomer cement (RMGIC). We mixed the RMGIC using a liquid-to-powder ratio (three drops of liquid:one scoop of powder) higher than that recommended by the manufacturer (one drop of liquid:one scoop of powder). The aim of using a high liquid proportion was to decrease the physical and mechanical properties of the RMGIC and help mimic the affected dentin. Furthermore, we mixed the RMGIC with a color modifier (resin staining) to produce the brownish appearance of the caries lesions. Another potential effect of a color modifier is reducing light penetration, which may subsequently limit light-activated polymerization to decrease the stiffness of the RMGIC [18]. A limitation of this study was the lack of a hardness assessment of the caries layer compared with the printed resin. The hardness value number (HVN) of a caries or affected dentin and sound dentin should be approximately 50–55 HVN and 100–105 HVN, respectively [19].

Although some limitations of the new 3D-printed teeth exist, most of the students still preferred the 3D-printed teeth for preclinical training. The results of the current study are also in agreement with previous studies that showed a positive response toward using 3D-printed teeth to practice caries removals [5,8,12]. Three-dimensional printed teeth can enhance clinical learning outcomes after training, which is similar to what is found with standard teeth [13]. We used a standard plastic tooth, which can be conveniently fixed in the standard model and a phantom head. The main limitation of this study is that the pulpal structures, such as the pulpal chamber, may not have been similar to real pupal structures. Furthermore, the remaining dentin thickness could have been decreased to mimic the clinical reality of deep caries lesions. These limitations can be addressed in future works.

In the current study, a single assessor assessed the outcome of the selective caries removals using the performance rating scale. Conventional visual assessment cavity preparations provide lower consistent intra-examiner reliability compared with digital assessments [20]. However, crude visual assessments are still commonly used in preclinical and clinical training. Additionally, digitally based assessment tools may provide effective skill training similar to that provided by a supervisor [21]. This could be beneficial in a situation where the student-to-faculty ratio is inadequate. The use of printed teeth may help increase the quality of caries removal teaching among dental students and thus reduce adverse effects, reduce treatment costs for patients, and increase the self-confidence of dental students [7]. The results of the current study showed that simulated deep caries can be prepared in house using commonly available dental materials. Future studies should investigate the potential use of augmented feedback from clinical tutors or the impact of 3D-scanned reports on the progression of the caries removal performance of students. This may help to promote enhanced learning outcomes in preclinical teaching and enhance self-learning in students.

## 5. Conclusions

We prepared 3D-printed teeth with added caries lesions and a pulp chamber for use in practicing the selective caries removal technique. Most students agreed that 3D-printed teeth should be employed for practice prior to treating real patients. Three-dimensional printed teeth could be considered an alternative learning tool for increasing students’ skills with the selective caries removal technique.

## Figures and Tables

**Table 1:** The materials used to prepare 3D-printed teeth to practice caries removal in the current study.

Materials	Functions	Suppliers	Lot Number
Resin-modified glass ionomer cements (Fuji II LC universal restorative)	Caries layer	GC Corporation, Bunkyoku, Tokyo, Japan	2105081
Color modifier (Kolor + Plus, Brown)	Caries layer	Kerr Corporation, Orange, CA, USA	8573172
Resin composite (Filtek Z350 XW Body Shade, 3M ESPE, USA)	Occlusal layer	3M, Saint Paul, MN, USA	7018XWB
Casting wax (red)	Dental pulp	Whip Mix, Louisville, KY, USA	6106201
3D printer resin (optiprint model)	3D-printed molars	Dentona AG, Dortmund, Germany	2111051

**Table 2:** Post-cavity-preparation questionnaire. We asked students to rate their agreement with the following items.

Items and Scores	Strongly Agree (5)	Agree (4)	Neutral (3)	Disagree (2)	Strongly Disagree (1)
1.1 The 3D-printed teeth are more realistic than plastic teeth when used for cavity preparation					
1.2 The 3D-printed teeth are more suitable for cavity preparation than plastic teeth					
1.3 The 3D-printed teeth are easy to handle and use compared with plastic teeth					
2.1 The 3D-printed teeth are more realistic than extracted teeth when used for cavity preparation					
2.2 The 3D-printed teeth are more suitable for cavity preparation than extracted teeth					
2.3 The 3D-printed teeth are easy to handle and use compared with extracted teeth					
3.1 The 3D-printed teeth help motivate you to practice the SCR technique compared with plastic and extracted teeth					
3.2 The 3D-printed teeth should have been used to practice the SCR technique before treating real patients					
3.3 The 3D-printed teeth help you practice basic fine finger movements					
3.4 The 3D-printed teeth help you increase tactile sensations and your skills with different instruments when using the SCR technique					

## Data Availability

The data presented in this study are available upon request to the corresponding author.
